# Current major depressive syndrome measured with the Patient Health Questionnaire-9 (PHQ-9) and the Composite International Diagnostic Interview (CIDI): results from a cross-sectional population-based study of adults in Germany

**DOI:** 10.1186/s12888-015-0463-4

**Published:** 2015-04-10

**Authors:** Ulrike E Maske, Markus A Busch, Frank Jacobi, Katja Beesdo-Baum, Ingeburg Seiffert, Hans-Ulrich Wittchen, Steffi Riedel-Heller, Ulfert Hapke

**Affiliations:** Department of Epidemiology and Health Monitoring, Robert Koch Institute, General-Pape-Straße 62-66, 12045 Berlin, Germany; Institute for Social Medicine, Occupational Health and Public Health, Faculty of Medicine, University of Leipzig, Ph.-Rosenthal-Str. 55, 04103 Leipzig, Germany; Institute of Clinical Psychology and Psychotherapy & Center for Clinical Epidemiology and Longitudinal Studies, Technische Universität Dresden, Chemnitzer Str. 46, 01187 Dresden, Germany; Behavioral Epidemiology, Technische Universität Dresden, Chemnitzer Str. 46, 01187 Dresden, Germany

**Keywords:** Depression, Patient health questionnaire, Composite international diagnostic interview, Population-based sample

## Abstract

**Background:**

Prevalence estimates for depression vary considerably by the type of assessment instrument, and there is limited information on their overlap in population-based samples. Our aim was to compare the Patient Health Questionnaire-9 (PHQ-9) with the Composite International Diagnostic Interview (CIDI) as measures for current major depressive syndrome (MDS) in a large population-based sample.

**Methods:**

Data derived from the mental health module of the nationwide cross-sectional German Health Interview and Examination Survey for Adults (DEGS1-MH) (n = 4483; age 18–79 years). MDS in the past two weeks was assessed (a) using the PHQ-9 diagnostic algorithm (PHQ-MDS) and (b) based on CIDI information about the latest symptom occurrence (recency) (CIDI-MDS). Prevalences, overall concordance and percentages of overlap of both MDS measures were determined. Prevalences of affirmed PHQ-9 depression symptoms and the mean and median PHQ-9 sum scores were analyzed per measure.

**Results:**

Prevalence of current MDS was 2.7% (95% CI: 2.0-3.6) for PHQ-MDS and 3.9% (95% CI: 3.1-5.0) for CIDI-MDS. The overall agreement between both measures was moderate (kappa: 0.43). Of all the participants, 1.5% (95% CI: 1.0-2.2) were classified as MDS cases by both measures, with 54.5% (95% CI: 42.7-65.9) of PHQ-MDS cases and 37.9% (95% CI: 27.8-49.1) of CIDI-MDS cases also being classified as MDS by the respective other MDS measure. However, 94.8% (95% CI: 93.6-95.8) of the participants were classified as non-MDS by both measures, with 97.5% (95% CI: 96.6-98.1) of non-PHQ-MDS and 98.7% (95% CI: 98.2-99.1) of non-CIDI-MDS being classified as non-MDS by the respective other MDS measure. The mean and median PHQ-9 sum score was higher in those with PHQ-MDS than in those with CIDI-MDS.

**Conclusions:**

Both measures have a high level of agreement for ruling out current MDS, but the overlap in their classification of cases is moderate. Our results indicate that they cannot be interpreted as equal measures of the same construct, suggesting limited comparability of their prevalence estimates. However, further exploration of algorithms and correlates and a proper labeling of measures in epidemiological studies are required.

## Background

Depressive disorders are a major public health issue with far reaching consequences for individuals and society [1,2]. Reliable and valid information at the population level is essential for estimating prevalences and associated care needs. Of the wide range of depressive categories defined in the Diagnostic and Statistical Manual of Mental Disorders (DSM-IV) [3], major depression is by far the most prevalent type in the general population [4]. In light of different research questions and mostly limited interview time, various instruments have been developed for mental health epidemiology and monitoring that differ in length, structure, and construct covered (i.e., at the level of symptoms, syndromes or the full clinical picture).

The Composite International Diagnostic Interview (CIDI) is an established and widely used instrument for assessing a clinical diagnosis of major depression in epidemiological and clinical studies [5-7]. It has been developed and validated for determining major depressive disorder (MDD) according to DSM-IV criteria in the past 12 months and over the lifetime, and major depressive syndrome (MDS), in which the exclusion criteria for MDD (i.e., mixed episodes, medical or substance-related reasons for symptoms, lifetime manic or hypomanic episodes) are disregarded. However, information about the latest occurrence, i.e., the recency of symptoms, is used in diverse studies based on the CIDI to determine a 4-week diagnosis [8-11]. The CIDI 4-week recency information for MDD was reported to be highly concordant with the diagnosis of MDD based on a professional psychiatric interview in a clinical sample [12], but the validity of recency information for determining a diagnosis for the past four weeks or shorter time frames has not yet been examined in the general population.

The Patient Health Questionnaire-9 (PHQ-9) is another established and frequently used instrument to assess MDS in clinical and population-based studies. Although it is also based on the DSM-IV criteria of major depression, the PHQ-9 has been specifically developed as a screening instrument for current depressive symptoms and syndromes in the past two weeks [13,14]. The PHQ-9 diagnostic algorithm has been extensively validated in clinical samples with varying results [15-17], but there are practically no studies on its diagnostic validity for determining current MDS with an appropriate reference instrument in the general population.

In this study, we use data from a large sample of the adult general population of Germany to explore the agreement and comparability of the PHQ-9 diagnostic algorithm and the CIDI information about symptom recency as measures for MDS in the past two weeks. Therefore, for both MDS measures, we investigated (1) the prevalence, (2) the percentages of overlap between these measures, and (3) the prevalence of single PHQ-9 depression symptoms and the mean and median PHQ-9 sum score. (4) Additionally, we examined the percentage of PHQ-MDS cases identified as 12-month MDS cases by the CIDI.

## Methods

### Study design and sample

Cross-sectional data of the mental health module (DEGS1-MH) of the German Health Interview and Examination Survey for Adults (DEGS1) (data collection: 2009–2012) were used. The study design and sample have been described elsewhere [18-20]. DEGS1 and DEGS1-MH included a representative sample of community-dwelling residents of Germany aged 18–79 years. In DEGS1-MH, 25 mental disorders were assessed with the DEGS-CIDI, a modified version of the German version (DIA-X/M-CIDI) [7,12] of the World Health Organization CIDI [5,20], complemented by additional questionnaires including the PHQ-9. Of the 8151 DEGS1 participants, 6027 were eligible for DEGS1-MH, and 5317 of these participated in the DEGS1-MH (conditional response rate: 88.2%) [20]. Of these, 4483 had full mental health interviews (one participant withdrew informed consent after publication of the study protocol), which were conducted by clinically trained interviewers, of which 94% were clinical psychologists or advanced clinical psychology students [20]. Participants with missing data for any of the measures of MDS were excluded from this study.

The DEGS1 study protocol was consented by the Federal and State Commissioners for Data Protection and was approved by the Charité-Universitätsmedizin Berlin ethics committee (No. EA2/047/08) and by the Ethics Board of the Technische Universität Dresden for DEGS1-MH (No. EK174062009). The participants gave written informed consent prior to the interviews.

### Measures of current MDS

#### PHQ-9 diagnostic algorithm

The self-administered German version of the PHQ-9 [21] was applied prior to the CIDI depression section. For each of the nine DSM-IV depression symptoms, the PHQ-9 assesses how often the respondent has been bothered by that symptom over the past two weeks, assigning values of 0 to 3 points (0 - not at all, 1 - several days, 2 - more than half of the days, 3 - nearly every day). According to Löwe et al., a case of current major depressive syndrome (PHQ-MDS) was determined when at least five symptoms were reported as present for more than half the days (suicide item: several days or more) in the past two weeks, including depressed mood or lack of interest [21].

#### 12-month and 2-week CIDI-MDS

In the DIA-X/M-CIDI, the standard algorithm aggregates thirty single items into the nine DSM-IV depression symptoms. MDS within the past 12 months is defined as at least five of the nine symptoms present most of the days for a minimum of two weeks, including depressed mood or lack of interest present most of the time of the day, disregarding additional information on exclusion criteria such as medication or substance use or bereavement, which is used in the definition of MDD [3]. As the 12-month time frame was not appropriate for assessing current MDS, a CIDI measure for current MDS was defined in analogy to the algorithm used to determine 4-week diagnoses of disorders [8-10,22]: MDS in the past two weeks (CIDI-MDS) was defined as 12-month MDS with recency of depressed mood or loss of interest, or tiredness or exhaustion, and “further depression symptoms” in the past two weeks.

### Statistical analysis

Prevalences with 95% confidence intervals (95% CIs) of PHQ-MDS and CIDI-MDS were estimated. Because of the lack of a gold standard for current MDS, operational characteristics (sensitivity and specificity) were not calculated, but the percentages of overlap between both measures were calculated. Concordance between the measures was examined using Cohen’s kappa [23]. Additionally, the percentage of PHQ-MDS cases identified as 12-month MDS cases according to the CIDI was examined. Finally, the prevalence of the single PHQ-9 depression symptoms present on more than half the days (suicide item: several days or more) in the past two weeks and the mean PHQ-9 sum scores by MDS measure were calculated. Sex- or age-specific results were not reported due to the small number of cases.

Differences were considered statistically significant if the 95% CIs did not overlap. Sample weights were used to account for participation probability and correcting sample deviations from population structure (as of Dec 31, 2010) in age, sex, region, nationality, type of municipality and education [20]. Therefore, all figures presented are weighted population estimates (except the median PHQ-9 sum score). To account for the clustering of participants within sample points, Stata 12.1 survey procedures were used.

## Results

Of the 4483 DEGS1-MH participants, 126 (2.8%) had missing values for the PHQ-MDS, 51 (1.1%) for the CIDI-MDS, and 24 (0.5%) for both measures, leaving a study sample of n = 4282. Of those, 50.8% (95% CI: 48.8-52.8) were female, the mean age was 47.8 years (95% CI: 47.2-48.4), 59.1% (95% CI: 56.7-61.5) were married, 34.5% (95% CI: 32.0-37.1) had a low education level, and 51.1% (95% CI: 48.9-53.2) had a medium education level according to the Comparative Analysis of Social Mobility in Industrial Nations (CASMIN) [24].

### Concordance and overlap of the PHQ-MDS and CIDI-MDS

Prevalence of current MDS was 2.7% (95% CI: 2.0-3.6) according to PHQ-MDS and 3.9% (95%CI: 3.1-5.0) according to CIDI-MDS (Table 1). Overall, 1.5% of participants (95% CI: 1.0-2.2) were classified as MDS cases and 94.8% as non-MDS cases by both instruments. The overall agreement between PHQ-MDS and CIDI-MDS was moderate (kappa: 0.43).

**Table 1:** **Prevalence of MDS and non**-**MDS according to PHQ**-**MDS** ^**1**^ **, CIDI**-**MDS** ^**2**^ **or both**

		**CIDI**-**MDS**		
		**MDS (n** = **130)**	**Non**-**MDS (n** = **4152)**	**Total**
**PHQ**-**MDS**	**MDS (n** = **86)**	1.5 (1.0-2.2)	1.2 (0.9-1.8)	2.7 (2.0-3.6)
	**Non**-**MDS (n** = **4196)**	2.4 (1.8-3.3)	94.8 (93.6-95.8)	97.3 (96.4-98.0)
	**Total**	3.9 (3.1-5.0)	96.1 (95.0-96.9)	100

^1^current major depressive syndrome according to the diagnostic algorithm of the Patient Health Questionnaire-9 (PHQ-9).

^2^2-week major depressive syndrome according to the CIDI information about symptom recency.

Of those with PHQ-MDS, 54.5% (95% CI: 42.7-65.9) were classified as CIDI-MDS (Table 2), and of those with CIDI-MDS, 37.9% (95% CI: 27.8-49.1) were classified as PHQ-MDS. The percentages of concordant classification as non-MDS cases were at least 97.4% in both directions.

**Table 2:** **Percentages of overlap of MDS and non**-**MDS cases according to PHQ**-**MDS** ^**1**^ **and CIDI**-**MDS** ^**2**^

	**% (95% CI)**
PHQ-MDS classified as CIDI-MDS	54.5 (42.7-65.9)
Non-PHQ-MDS classified as non-CIDI-MDS	97.5 (96.6-98.1)
CIDI-MDS classified as PHQ-MDS	37.9 (27.8-49.1)
Non-CIDI-MDS classified as non-PHQ-MDS	98.7 (98.2-99.1)

^1^current major depressive syndrome according to the diagnostic algorithm of the Patient Health Questionnaire-9 (PHQ-9).

^2^2-week major depressive syndrome according to the CIDI information about symptom recency.

Of those with PHQ-MDS, 78.8% (95% CI: 67.6-86.72) were classified as 12-month CIDI-MDS cases.

### PHQ-9 depression symptoms and PHQ-9 sum score

Overall, the prevalence of all the PHQ-9 depression symptoms except “psychomotor retardation or agitation” was higher among participants with PHQ-MDS compared to those with CIDI-MDS (Table 3). CIDI-MDS cases were less likely than PHQ-MDS cases to affirm depressed mood and lack of interest as main depression symptoms in the PHQ-9. Additional analyses showed that 58.9% (95% CI: 47.9-69.1) of those with CIDI-MDS affirmed at least one of these two symptoms. CIDI-MDS cases had a lower mean and median PHQ-9 sum score (11.7 and 10.5) than PHQ-MDS cases (15.9 and 15).

**Table 3:** **Prevalence of PHQ**-**9 depression symptoms and mean**/**median PHQ**-**9 score among participants with PHQ**-**MDS** ^**1**^ **and CIDI**-**MDS** ^**2**^

	**PHQ-MDS % (95% CI) n = 86**	**CIDI-MDS % (95% CI) n = 130**
**PHQ**-**9 depression symptoms present more than half of the days**		
Little interest or pleasure in doing things	68.7 (54.1-80.3)	38.6 (28.5-49.8)
Feeling down, depressed, or hopeless	77.1 (63.0-87.0)	42.5 (31.6-54.3)
Trouble falling or staying asleep, or sleeping too much	83.5 (71.4-91.1)	60.9 (49.6-71.1)
Feeling tired or having little energy	97.7 (89.9-99.5)	68.8 (57.5-78.3)
Poor appetite or overeating	72.8 (60.7-82.2)	41.9 (31.3-53.2)
Feeling bad about yourself or that you are a failure or have let yourself or your family down	72.1 (59.6-81.9)	39.4 (29.1-50.8)
Trouble concentrating on things, such as reading the newspaper or watching television	52.2 (39.3-64.8)	34.5 (24.2-46.5)
Moving or speaking so slowly that other people could have noticed, or the opposite — being so fidgety or restless that you have been moving around a lot more than usual	17.7 (10.5-28.3)	8.3 (4.3-15.3)
Thoughts that you would be better off dead or of hurting yourself in some way^3^	61.0 (46.8-73.6)	41.9 (32.1-52.5)
Mean PHQ-9 score	15.9 (14.9-17.0)	11.7 (10.6-12.8)
Median PHQ-9 score	15	10.5

^1^current major depressive syndrome according to the diagnostic algorithm of the Patient Health Questionnaire-9 (PHQ-9).

^2^2-week major depressive syndrome according to the CIDI information about symptom recency.

^3^present at least “several days”.

## Discussion

This study compares two measures of current MDS in the past two weeks based on the PHQ-9 diagnostic algorithm and on information of symptom recency from the CIDI in a large population-based sample of adults in Germany. Prevalence was 2.7% for PHQ-MDS and 3.9% for CIDI-MDS. Our analyses showed a moderate overall concordance of both, with a high agreement regarding the classification as non-MDS, but with moderate agreement in their classification as MDS. CIDI-MDS cases had lower prevalences of single PHQ-9 items affirmed and a lower mean PHQ-9 sum score than those with PHQ-MDS. More than three-quarters of those with PHQ-MDS were included in the 12-month CIDI-MDS diagnosis.

The difference in prevalence is plausible considering that the PHQ-9 focuses on the narrow time frame of two weeks before the assessment, whereas the 2-week CIDI-MDS comprises those cases of any episode of MDS in the past 12 months, who reported the past two weeks as the latest presence of depressed mood or loss of interest or tiredness/exhaustion and “some further symptoms”.

The moderate percentage of overlap between PHQ-MDS and CIDI-MDS in the past two weeks appears remarkable at first sight considering that both instruments cover the same time frame of the past two weeks and are based on the criteria of the DSM-IV. In our data, 37.9% of those with CIDI-MDS were also classified as PHQ-MDS, which is rather low compared to studies in clinical samples with the Structured Clinical Interview for DSM-IV Disorders (SCID) as the reference instrument [14,16]. However, there are several plausible explanations for this finding. First, depression is likely to be less frequent in the general population than in clinical samples, and the ability of the PHQ-9 to detect MDS has been questioned in samples with low pretest probabilities [16]. Second, milder forms of depression are likely to be more common in a general population sample than in a clinical sample, and the agreement of diagnostic instruments may be lower in such samples [25-27].

Two further explanations for the discrepancy are possible, resulting from differences in the assessment of symptom severity and in the algorithms applied in the measures examined. Regarding the PHQ-9, the wording concerning the temporal pattern of the occurrence of symptoms is slightly less stringently specified than in the DSM-IV in that it demands five of nine symptoms to be present at least half of the days in the past two weeks; however, it does not specify the presence as “most of the time of the day” for the main symptoms, as does the DSM-IV [3] and the CIDI. Thus, PHQ-MDS is likely to include cases whose main symptoms were potentially less severe than demanded in the DSM-IV, which might also explain why 78.8% and not all of those with PHQ-MDS were identified as 12-month CIDI-MDS cases.

Regarding the CIDI, the presence of the full MDS criteria is assessed for at least two weeks in the past twelve months but not necessarily for the time indicated in the question about the latest symptom occurrence. To determine the recency, potential 12-month MDS cases were asked to indicate the latest occurrence of depressed mood or loss of interest or additional tiredness/exhaustion and “some further symptoms”. First, this might lead to a classification of current MDS in respondents with subthreshold depression symptoms, which is supported by the finding that the mean and median PHQ-9 sum scores for 2-week CIDI-MDS were rather low (11.7 and 10.5) and that the PHQ-9 items on depressed mood and loss of interest as main depression symptoms were reported substantially less frequently than in those with PHQ-MDS. Second, 2-week CIDI-MDS does not include depressed mood and lack of interest as the only main symptoms; it also includes loss of interest following the ICD-10 [28]. This difference between the algorithms of PHQ-MDS and 2-week CIDI-MDS might be an additional explanation for the discrepancies found.

The fact that 12-month CIDI-MDS cases do not necessarily experience the full symptomatic picture in the two weeks prior to the interview is self-evident considering the episodic course of depression, i.e., the variation of symptom severity between the full clinical picture and subclinical symptoms [29,30] or partial remission with or without treatment.

### Limitations

Several limitations must be considered. Firstly, there is limited information about the diagnostic validity of PHQ-MDS and 2-week CIDI-MDS based on the information about symptom recency in general population samples; therefore, neither can be considered the gold standard. Secondly, symptom assessments and algorithms differ between the measures investigated, confining the options of identification of reasons for discrepancy. Thirdly, because the definition of MDS does not comprise differential diagnosis, MDS according to both measures might occur in the context of other mental disorders, e.g., bipolar disorder. Fourthly, because cases with severe depression symptoms are less likely to participate in health surveys, reported prevalences are presumably conservative with regard to the prevalence of MDS in the general population in Germany aged 18–79 years.

## Conclusion

The results suggest that both the PHQ-9 diagnostic algorithm and CIDI-MDS in the past two weeks may be useful to rule out current MDS in samples of the general population. The PHQ-9 may be more useful for general population surveys with limited interview time because of its brevity, flexible use as a diagnostic algorithm and severity score, different validated assessment modes and frequent worldwide use. Nevertheless, one should consider that the diagnostic algorithm might include depression cases with less persistent symptoms in the current episode than are defined in the DSM-IV. Thus, its validity in screening for or determining current MDS in the general population must be further examined with an appropriate reference instrument in a large sample, analyzing possible sex- and age-specific differences as well. When using the CIDI recency information as an indicator for the latest clinical diagnosis, researchers should keep in mind and explicitly discuss that it might include a considerable number of subthreshold cases, particularly in general population samples. Our findings raise the question of whether this is also the case with regard to other time frames based on the recency information. This question has yet to be investigated with a valid gold standard to determine to what extent recency information can be interpreted as a valid indicator for a clinical diagnosis. Obviously, this does not affect the CIDI 12-month perspective, which remains the gold standard for determining depressive disorders in the past 12 months, even more so because it also allows determining MDD diagnosis with exclusion criteria.

The fact that we found only moderate overlap of the two measures for current MDS, which have both been developed based on the same criteria, supports other research showing that even minor wording changes in depression measures may lead to major changes in prevalence estimates [31]. Thus, it is important for researchers in the field of depression to carefully describe and precisely discuss the constructs and time frames covered by specific depression measures. Additionally, it is important to use adequate terminology for each depression measure instead of subsuming any measures under the general name “depression”.

## Abbreviations

DSM-IV: Diagnostic and statistical manual of mental disorders, 4th edition
CIDI: Composite international diagnostic interview
MDD: Major depressive disorder
MDS: Major depressive syndrome
PHQ-9: Patient health questionnaire-9
DEGS1-MH: Mental health module of the German Health Interview and Examination Survey for Adults (DEGS1)
95% CI: 95% confidence interval
SCID: Structured clinical interview for DSM-IV disorders
ICD-10: International statistical classification of diseases and related health problems
